# Transcriptomic Profiling of Differential Responses to Drought in Two Freshwater Mussel Species, the Giant Floater *Pyganodon grandis* and the Pondhorn *Uniomerus tetralasmus*

**DOI:** 10.1371/journal.pone.0089481

**Published:** 2014-02-25

**Authors:** Yupeng Luo, Chao Li, Andrew Gascho Landis, Guiling Wang, James Stoeckel, Eric Peatman

**Affiliations:** 1 School of Fisheries, Aquaculture, and Aquatic Sciences, Auburn University, Auburn, Alabama, United States of America; 2 Key Laboratory of Freshwater Aquatic Genetic Resources, Shanghai Ocean University, Ministry of Agriculture, Shanghai, People's Republic of China; 3 Georgia Department of Natural Resources, Wildlife Resources Division, Nongame Conservation Section, Social Circle, Georgia, United States of America; University of Connecticut, United States of America

## Abstract

The southeastern US has experienced recurrent drought during recent decades. Increasing demand for water, as precipitation decreases, exacerbates stress on the aquatic biota of the Southeast: a global hotspot for freshwater mussel, crayfish, and fish diversity. Freshwater unionid mussels are ideal candidates to study linkages between ecophysiological and behavioral responses to drought. Previous work on co-occurring mussel species suggests a coupling of physiology and behavior along a gradient ranging from intolerant species such as *Pyganodon grandis* (giant floater) that track receding waters and rarely burrow in the substrates to tolerant species such as *Uniomerus tetralasmus* (pondhorn) that rarely track receding waters, but readily burrow into the drying sediments. We utilized a next-generation sequencing-based RNA-Seq approach to examine heat/desiccation-induced transcriptomic profiles of these two species in order to identify linkages between patterns of gene expression, physiology and behavior. Sequencing produced over 425 million 100 bp reads. Using the *de novo* assembly package Trinity, we assembled the short reads into 321,250 contigs from giant floater (average length 835 bp) and 385,735 contigs from pondhorn (average length 929 bp). BLAST-based annotation and gene expression analysis revealed 2,832 differentially expressed genes in giant floater and 2,758 differentially expressed genes in pondhorn. Trancriptomic responses included changes in molecular chaperones, oxidative stress profiles, cell cycling, energy metabolism, immunity, and cytoskeletal rearrangements. Comparative analyses between species indicated significantly higher induction of molecular chaperones and cytoskeletal elements in the intolerant *P. grandis* as well as important differences in genes regulating apoptosis and immunity.

## Introduction

As drought conditions recur with increasing frequency and severity in the Southeastern U.S., sessile aquatic organisms in freshwater ecosystems, particularly in streams, are bearing the brunt of these environmental perturbances [1]–[3]. Freshwater unionid mussel populations are already among the most endangered groups of organisms in the world [4]. Growing efforts to document the stunning diversity and varied life history strategies of unionids are also recording the measurable impacts of altered stream flows and thermal profiles on survival, recruitment, reproductive strategies, and community structure of these species [2], [3], [5]–[7].

The ability of individual species to tolerate drought conditions depends on several factors including severity and duration of the disturbance, stream habitat (debris, pools, etc.), and differences in behavioral and physiological responses to emersion/desiccation and heat stress [3]. Indeed, a recent study utilizing field and laboratory experiments revealed links between behavioral responses, physiological tolerances, and survival in three co-existing mussel species (*Uniomerus tetralasmus* (pondhorn); *Pyganodon grandis* (giant floater); and *Lampsilis straminea* (fatmucket)). There, the authors observed that a burrowing response in pondhorn was correlated with a higher tolerance to desiccation and higher survival (77%) compared to the water-tracking behavior and low tolerance and survival (0%) of giant floater [3].

The identification of underlying genetic mechanisms regulating these behavioral and physiological differences would provide key insights into adaptive responses of freshwater mussels to heat stress and drought. Previous studies in marine shellfish species have explored connections between variations in gene and protein expression and differences in latitudinal adaptation, differential success of native and invasive species, and resistance to summer mortality in the context of heat stress [8]–[13]. While such studies have traditionally required time-consuming and expensive generation of molecular resources and have, therefore, been limited to a handful of key species, new RNA-Seq approaches have dramatically expanded our ability to carry out transcriptome profiling in non-model species [14]. Indeed, using RNA-Seq we recently identified the components of a classical heat stress response in the unionid *Villosa lienosa* [15]. Of even greater interest than highly conserved stress factors and responses [16], however, may be the identification of divergent responses and gene components accounting, at least in part, for heightened drought tolerance in some species or populations. Recent sequencing of the Pacific oyster genome revealed a startling diversity of genes available for adaptation to environmental stress, including 88 heat shock protein 70 (HSP70) genes (compared to 17 in humans) and 48 inhibitor of apoptosis (IAP) genes (compared to 8 in humans). The finding not only highlights the importance of these gene families in molluscs but also emphasizes the need for global expression profiling of particular species of interest rather than examination of a conserved, narrow subset of heat shock genes based on mammalian paradigms. Here, we conducted RNA-Seq-based transcriptome profiling on *P. grandis* and *U. tetralasmus* exposed to experimental heat stress/desiccation. Our main objectives were: (i) to compare the ability of Trinity and Trans-ABySS to assemble high quality transcriptomes for both species; (ii) identify key shared and divergent responses to drought in unionid mussels; and (iii) determine whether there were consistent differences between the tolerant and sensitive unionid species in both individual gene and pathway level responses to drought stress. The information gained here may serve as a foundation to guide natural resource managers in assessing the adaptive potential of freshwater mussel species in the face of climate change and should aid in the development of molecular tools to monitor stress levels of mussels in drought-stricken streams.

## Materials and Methods

### Experimental animal and tissue sampling

Two species of freshwater mussel, *P. grandis* and *U. tetralasmus*, were utilized in experiments. *Pyganodon grandis* have been designated a species of lowest conservation concern, and *U. tetralasmus* a species of moderate conservation concern in Alabama. *Uniomerus tetralasmus* were collected from Opintlocco Creek, located in the Tallapoosa catchment of east central Alabama, with permission from the J. W. Huskey family and under scientific collection permits from the Alabama Department of Conservation and Natural Resources. *Pyganodon grandis* were produced in experimental ponds at the South Auburn Fisheries Research Station, Auburn University as part of previous experiments by the Crustacean and Molluscan Ecology Lab. Prior to experiments, all mussels were submerged in a large water bath and maintained at 21°C. Pond water was used throughout the study so mussels could feed on natural food sources. All mussels were placed individually in 500 mL plastic cups within the water bath. Each cup was filled with sand and mussels were allowed to position themselves naturally in the substrate. After three days of acclimation at 21°C, temperature was increased 3°C/d over 4 d to 33°C for the experimental mussels, while temperature (21°C) and water level remained constant for the duration of the experiment for the control mussels. Upon reaching 33°C, mussels were maintained at that temperature for 2 d. On day 3 at 33°C, the water level in the water bath was lowered below the top of the cups and the water in each cup was drained.

At 24, 48, and 72 h after initiation of desiccation/emersion, non-lethal foot tissue samples were collected from 15 individuals per species using biopsy forceps. Mussels were randomly assigned to sampling time groups. After the 72 h samples were collected, the recovery phase of the experiment began. The remaining experimental mussels were submerged and temperatures were dropped to 21°C by 3°C/d for 4 d. After one day at 21°C, 15 mussels per species were sampled (5 d recovery timepoint). Tissues were flash frozen with liquid nitrogen immediately upon collection and stored in a −80°C freezer until RNA extraction.

### RNA extraction, library construction and sequencing

From each timepoint/treatment group, three replicate pools of 5 individual samples/pool were constructed. Pooled tissue samples were ground to a fine powder in the presence of liquid nitrogen. RNA was extracted from ground tissue using an RNeasy Kit (Qiagen, Valencia, California) following the manufacturer's instruction. RNA concentration and integrity of each sample were measured on an RNA 6000 Nano Bioanalysis chip using the Agilent Bioanalyzer 2100 on the RNA 6000 Nano Bioanalysis chip, only the samples with R.I.N. value >8 were used for the sequencing.

The control group (submerged 21°C) and 72 h (desiccation 33°C) experimental groups from each species were selected for RNA-Seq library construction, Illumina sequencing and expression analysis.

RNA-Seq library preparation and sequencing were carried out by HudsonAlpha Genomic Services Lab (Huntsville, AL, USA). cDNA libraries were constructed with 2.14–3.25 ug of starting total RNA and utilized the IlluminaTruSeq RNA Sample Preparation Kit (Illumina), as detailed in the TruSeq protocol. The libraries were amplified with 15 cycles of PCR and contained TruSeq indexes within the adaptors, specifically indexes 1–12. Finally, amplified library yields were 30 ul of 19.8–21.4 ng/ul with an average length of ∼270 bp, indicating a concentration of 110–140 nM. After KAPA quantitation and dilution, the libraries were clustered 12 per lane and sequenced on an Illumina HiSeq 2000 instrument with 100 bp PE read chemistry.

### *De novo* assembly of sequencing reads

The *de novo* assembly of short reads was performed on the mussel short reads using both ABySS and Trinity [17], [18], versions 1.3.2 and the 2012-10-05 editions, respectively. Before assembly, raw reads were trimmed by removing adaptor sequences and ambiguous nucleotides. Reads with quality scores less than 20 and length below 30 bp were all trimmed. The resulting high-quality sequences were used in the subsequent assembly.

In ABySS, briefly, the clean reads were first hashed according to a predefined k-mer length, the ‘k-mers’. After capturing overlaps of length k-1 between these k-mers, the short reads were assembled into contigs. The k-mer size was set from 50 to 96, assemblies from all k-mers were merged into one assembly by Trans-ABySS (version 1.4.4).

In Trinity, briefly, the raw reads were assembled into the unique sequences of transcripts in Inchworm via greedy k-mer extension (k-mer 25). After mapping of reads to Inchworm contigs, Chrysalis incorporated reads into de Bruijn graphs and the Butterfly module processed the individual graphs to generate full-length transcripts.

In order to reduce redundancy, the assembly results from different assemblers were passed to CD-Hit version 4.5.4 [19] and CAP3 [20] for multiple alignments and consensus building. The threshold was set as identity equal to 1 in CD-Hit, the minimal overlap length and identity equal to 100 bp and 99% in CAP3.

### Gene annotation and ontology

All contigs in the resulting Trinity assembly were used as queries for BLASTX searches against the UniProtKB/SwissProt database and the National Center for Biotechnology Information (NCBI) non-redundant (nr) protein database. The cutoff Expect value (E-value) was set at 1e−5 and only the top hit result was assigned as the annotation for each contig. Gene ontology (GO) annotation analysis was processed using the UniProtKB/SwissProt results in Blast2GO (version 2.5.0), which is an automated tool for the assignment of gene ontology terms. The final annotation file was generated after gene-ID mapping, GO term assignment, annotation augmentation and generic GO-Slim processes and categorized with regard to Biological Process, Cellular Component and Molecular Function.

### Identification of differentially expressed contigs

Differentially expressed contig analysis was performed using the RNA-Seq module and the expression analysis module in CLC Genomics Workbench. The high quality reads from each sample were mapped onto the Trinity reference assembly using CLC Genomics Workbench software. At least 95% of the bases were required to align to the reference and no more than two mismatches were allowed. The total mapped reads number for each transcript was determined and then normalized to detect RPKM (Reads Per Kilobase of exon model per Million mapped reads). After scaling normalization of the RPKM values, fold changes were calculated [21]. The proportions-based test was used to identify differentially expressed contigs between the control group and heat/desiccation group with three replications in each group with the threshold for significant differences set at a corrected p-value of *p*<0.05 [22]. Transcripts with absolute fold change values greater than 1.5 were defined as differentially expressed genes, which were divided into up- and down- regulated groups for further analysis.

Differentially expressed contigs were also used as queries for BLASTX searches against the *Crassostrea gigas* (Pacific oyster) protein database from NCBI with the cutoff Expect value (E-value) of 1e−5. Contigs from *P. grandis* and *U. tetralasmus* with shared annotation (nr and/or *C. gigas*) based on BLAST analysis were subjected to additional analyses to establish orthology including reciprocal BLAST, sequence alignment, and phylogenetic analysis. Differentially expressed contigs from a given species with no clear orthologous gene(s) in the other species were regarded here as unique.

### Gene ontology and enrichment analysis

GO analysis and enrichment analysis between significantly expressed GO terms and overall reference assembly GO terms was processed to select overrepresented GO annotations in the differentially expressed genes using Ontologizer 2.0 using the Parent-Child-Intersection method with a Benjamini-Hochberg multiple testing correction [23], [24]. GO terms for each gene were obtained by utilizing UniProtKB/SwissProt database annotations for the unigene set. The difference in the frequency of GO terms annotation in the differentially expressed genes sets were compared to the overall mussel reference assembly for each species. The threshold was set as FDR value <0.1.

Functional groups and pathways encompassing the differentially expressed genes were identified based on GO analysis, pathway analysis based on the Kyoto Encyclopedia of Genes and Genomes (KEGG) database, and manual literature review. Based on these analyses, key genes were organized into broad functional categories. Fold change differences between species for a given gene in each category were assessed for significance using a simple t-test on log-transformed RPKM values. Category-level differences in fold changes between species were assessed using a paired t-test.

### Experimental validation—QPCR

Sixteen significantly expressed genes, including 7 shared genes, 4 unique genes of *P. grandis* and 5 unique genes of *U. tetralasmus*, with different expression patterns were selected for validation of the RNA-Seq results using real time QPCR. Beta-actin was set as the reference gene in both species. Primers were designed based on contig sequences using Primer Premier 6 (Table S1). Tissue samples from the 72 h experimental group and the control group were used as the template for QPCR. cDNA was synthesized using the iScript cDNA Synthesis Kit (Bio-Rad) according to manufacturer's protocol. The iScript chemistry uses a blend of oligo-dT and random hexamer primers. All the cDNA products were diluted to 250 ng/µl and utilized for the quantitative real-time PCR reactions using the SsoFast EvaGreen Supermix on a CFX96 real-time PCR Detection System (Bio-Rad Laboratories, Hercules, CA). The thermal cycling profile consisted of an initial denaturation at 95°C (for 30 s), followed by 40 cycles of denaturation at 94°C (5 s), and an appropriate annealing/extension temperature (58°C, 5 s). An additional temperature ramping step was utilized to produce melting curves of the reaction from 65°C to 95°C. Results were expressed relative to the expression levels of beta actin in each sample using the Relative Expression Software Tool (REST) version 2009 [25]. The biological replicate fluorescence intensities of the control and treatment products for each gene, as measured by crossing-point (Ct) values, were compared and converted to fold differences by the relative quantification method. Expression differences between groups were assessed for statistical significance using a randomization test in the REST software. The mRNA expression levels of all samples were normalized to the levels of beta actin gene in the same samples. Test amplifications were conducted with ensure that beta actin and target genes were within an acceptable range. A no-template control was run on all plates. QPCR analysis was repeated in triplicate runs (technical replicates) to confirm expression patterns.

### Temporal expression analysis of key genes

A subset of key classical heat shock proteins and species-specific genes differentially expressed at 72 h were selected to examine their broader transcriptional profiles. Primers, reference gene, and reaction conditions were the same as described above. Relative expression profiles were captured for the experimental samples of each species at the 24 h, 48 h, and 5 d recovery timepoints. Data were analyzed as described above.

## Results

### Sequencing of short expressed reads from mussel foot tissue

Illumina-based RNA-sequencing (RNA-Seq) was carried out on RNA extracted from replicated, pooled foot tissue samples from *P. grandis* and *U. tetralasmus* exposed to control and heat stress/desiccation (72 h) experimental conditions. Reads from different samples were distinguished through the use of multiple identifier (MID) tags. A total of 427.5 million reads were generated on an Illumina HiSeq2000 instrument, including 201.1 million reads from *P. grandis*, and 226.4 million reads from *U. tetralasmus* (Table S2). At least 29 million reads were generated from each barcoded sample with an average of 35.6 million reads/library. Raw data was archived at the NCBI Sequence Read Archive (SRA) under Accession **SRP026193**.

### *De novo* assembly of mussel transcriptomes

*De novo* assembly of RNA-Seq reads can be achieved using several assembly algorithms and software programs. We had previously developed an in-house bioinformatics pipeline around Trans-ABySS and demonstrated superior performance relative to other assembly options in several species including freshwater mussel [15], [26]–[28]. However, the recently developed Trinity software package provides several potential advantages for transcriptome profiling in non-model species [18]. Therefore, here we sought to compare critical metrics of performance in transcriptome assembly between Tran-ABySS and Trinity.

#### Trans-ABySS

From *P. grandis* cleaned reads, Trans-ABySS generated 804,906 contigs including 178,956 contigs longer than 1000 bp. Average contig length was 772.6 bp while N50 was 801 bp. After filtration of redundant contigs by CD-Hit and CAP3, 56.6% of total contigs remained (455,659) with average length of 616.0 bp. The percentages of reads mapped in pairs and mapped to the final reference were 62.8% and 83.7%, respectively (Table 1).

**Table 1:** Summary of *de novo* assembly results of Illumina cleaned reads from *P. grandis* and *U. tetralasmus* foot tissue using Trans-ABySS and Trinity.

	*P. grandis*		*U. tetralasmus*	
	Trans-ABySS	Trinity	Trans-ABySS	Trinity
Contigs	804,906	336,799	499,413	405,996
Large contigs (≥1000 bp)	178,956	66,959	147,046	81,095
N50 (bp)	801	2,100	1,503	2,233
Average contig length	772.6	970.1	963.0	968.1
Contigs (After CD-HIT-EST+ CAP3)	455,659	321,250	261,489	385,735
Percentage contigs kept after redundant removing	56.6%	95.4%	52.4%	95.0%
Average length (bp) (After CD-HIT-EST+ CAP3)	616.0	835.0	885.5	929.3
Reads mapped in pairs (%)	62.8	79.2	65.9	79.5
Reads mapped to final reference (%)	83.7	84.5	82.0	83.8

From *U. tetralasmus* cleaned reads, Trans-ABySS generated 499,413 contigs including 147,046 contigs longer than 1000 bp. Average contig length was 963.0 bp while N50 was 1,503 bp. After filtration of redundant contigs by CD-Hit and CAP3, 52.4% of total contigs remained (261,489) with average length of 885.5 bp. The percentages of reads mapped in pairs and mapped to the final reference were 65.9% and 82.0%, respectively (Table 1).

#### Trinity

From *P. grandis* cleaned reads, Trinity generated 336,799 contigs including 66,959 contigs longer than 1,000 bp. Average contig length was 970.1 bp while N50 was 2,100 bp. After filtration of redundant contigs by CD-Hit and CAP3, 95.4% of total contigs remained (321,250) with average length of 835.0 bp. The percentages of reads mapped in pairs and mapped to the final reference were 79.2% and 84.5%, respectively (Table 1).

From *U. tetralasmus* cleaned reads, Trinity generated 405,996 contigs including 81,095 contigs longer than 1,000 bp. Average contig length was 968.1 bp while N50 was 2,233 bp. After filtration of redundant contigs by CD-Hit and CAP3, 95.0% of total contigs remained (385,735) with average length of 929.3 bp. The percentages of reads mapped in pairs and mapped to the final reference were 79.5% and 83.8%, respectively (Table 1).

#### Best assembly selection

Comparison of the assemblies generated by Tran-ABySS and Trinity (Table 1) made clear that, although Tran-ABySS generated a larger initial count of contigs, redundancy was much higher than observed with Trinity. CD-HIT/CAP3 trimmed over 40% of Trans-ABySS contigs compared with less than 5% of Trinity contigs. Additionally, Trinity produced contigs with greater N50, average length and mapped reads metrics. Trinity was able to assemble transcript pieces into longer contigs with average lengths over 200 bp greater than Trans-ABySS and a high rate of paired read mapping. Considering these results, we utilized the Trinity assembly for subsequent analysis. Trinity contig assemblies for *P. grandis* and *U. tetralasmus* are available upon request.

### Gene identification and annotation

Trinity contigs were used to query the UniProtKB/SwissProt and NCBI non-redundant (nr) protein databases by BLASTX (Table 2).

**Table 2:** Summary of gene identification and annotation of assembled mussel contigs based on BLAST homology searches against various protein databases (UniProt, nr).

	*P. grandis*		*U. tetralasmus*	
	UniProt	NR	UniProt	NR
Contigs with putative gene matches	48,448	57,469	56,170	66,381
Percentage of annotated contigs	14.38%	17.06%	13.84%	16.35%
Annotated contigs ≥500 bp	28,205	32,290	33,595	38,375
Annotated contigs≥1000 bp	11,773	13,243	14,391	16,017
Unigene matches	15,045	22,616	15,170	20,610
Hypothetical gene matches	0	6,120	0	6,101
Quality Unigenematches	11,278	11,877	11,324	10,699

Putative gene matches were at E-value ≤ 1e−5. Hypothetical gene matches denote those BLAST hits with uninformative annotation. Quality unigene hits denote more stringent parameters, including score≥100, E-value ≤ 1e−20.

In *P. grandis*, 57,469 contigs had significant BLASTX hits against 22,616 unique nr proteins. Of these, 6,120 contigs had top BLAST hits against hypothetical gene matches (predicted proteins and/or those with no annotation). Additionally, 11,877 unigenes could be identified based on more stringent criteria of a BLAST score ≥100 and E-value ≤ 1e−20 (quality matches). The same BLAST criteria were used to query the UniProt database.

In *U. tetralasmus*, 66,381 contigs had significant BLASTX hits against 20,610 unique nr proteins. Of these, 6,101 contigs had top BLAST hits against hypothetical gene matches (predicted proteins and/or those with no annotation). Additionally, 10,699 unigenes could be identified based on more stringent criteria of a BLAST score ≥100 and E-value ≤ 1e−20 (quality matches). The same BLAST criteria were used to query the UniProt database.

### Identification and analysis of differentially expressed genes

Differential expression analyses in comparison to control samples were carried out for both the *P. grandis* and *U. tetralasmus* experimental samples (Table S3; Table 3). In *P. grandis*, a total of 2,559 genes (unique annotated contigs with significant nr database BLASTX identities) including 1,499 up-regulated genes and 1,060 down-regulated genes were differentially expressed (≥1.5-fold, corrected p-value <0.05) following 72 h of exposure to heat and desiccation stress. Similarly, in U. tetralasmus, 2,532 genes including 1,594 up-regulated genes and 938 down-regulated genes were differentially expressed. Read coverage, critical in accurate determination of fold change, averaged 797 and 567 reads/contig for *P. grandis* and *U. tetralasmus*, respectively. We also used the differentially expressed contigs of both species as queries against annotated proteins from the recently sequenced Pacific oyster, *C. gigas* genome (the only publicly available genome among bivalve species [29]). Annotating based on hits to the *C. gigas* protein database identified 1,863 differentially expressed genes in *P. grandis* and 1,744 differentially expressed genes in *U. tetralasmus*.

**Table 3:** Statistics of differentially expressed genes of *P. grandis* and *U. tetralasmus* annotated by nr and *C. gigas* protein database following drought challenge.

	*P. grandis*		*U. tetralasmus*	
	NR	*C. gigas*	NR	*C. gigas*
Up-regulated	1,499	1,093	1,594	1,126
Down-regulated	1,060	770	938	648
Total unigenes	2,559	1,863	2,532	1,774
Reads per contig	797	718	569	586

Values indicate contigs/genes passing cutoff values of fold change ≥1.5 (corrected p<0.05) and read number per contig ≥5. Reads/contig refers to average contig size.

While fewer contigs were annotated by *C. gigas*, its larger, comprehensive protein database aided in initial identification of “shared” genes present in sets of differentially expressed genes from both mussel species. For example, we identified 318 and 380 significantly up-regulated contigs with the same top hit in both species using nr and *C. gigas* annotation, respectively. Similarly, 190 contigs shared the same top hit and were down-regulated in both species using *C. gigas* annotation. Finally, there are 130 and 162 differentially expressed genes in each species shared the same BLAST identity but showed fold changes in opposite directions.

### Enrichment and pathway analysis

Gene ontology (GO) annotations were assigned using Blast2GO. In *P. grandis*, 7,571 GO terms were assigned to 2,559 unique genes including 2,660 (35.1%) cellular component terms, 1,629 (21.5%) molecular functions terms and 3,282 (43.4%) biological process terms. In *U. tetralasmus*, a total of 6,764 GO terms were assigned to 2,532 unique gene matches including 2,478 (36.6%) cellular component terms, 1530 (22.6%) molecular functions terms and 2,756 (40.8%) biological process terms. The percentages of annotated sequences of the two species assigned to GO terms are shown in Figure S1.

The differently expressed unique genes were then used as inputs to perform enrichment analysis using Ontologizer. Parent-child GO term enrichment analysis was performed to detect significantly overrepresented GO terms. A total of 23 and 50 overrepresented terms with *p* (FDR-corrected) <0.1 were detected in *P. grandis* and *U. tetralasmus*, respectively. Potentially informative higher level GO terms were carried for further pathway analysis. These included indications of changes in protein folding in both species, and species-specific enrichment of cellular transport, cell structure and energy metabolism in *P. grandis* and genes negatively regulating cell death and the immune response in *U. tetralasmus* (Table S4). However, the general lack of functionally annotated genes in molluscan species limited the extent of GO analysis that could be carried out.

Based on enrichment analysis, manual annotation, and literature searches, representative key genes were organized into 7 functional categories encompassing important shared and potentially species-specific response signatures to heat stress/desiccation (Table 4). These included chaperone/heat shock proteins, antioxidant/oxidative stress response, cell proliferation/apoptosis, energy metabolism, immune, cytoskeletal and uncategorized (other). While imputed putative functional roles of these genes are covered in-depth below (Discussion), several general trends were apparent. First, while a robust heat-shock response was mounted in both species, the drought-susceptible *P. grandis* manifested significantly higher and broader up-regulation of molecular chaperones at 72 h (p = 0.0277, paired t-test). Second, significantly different category-level fold changes were observed in genes regulating cytoskeletal arrangements (p = 0.0149), with higher up-regulation of cell fiber elements observed for *P. grandis*. Third, individual key genes within each category had significantly different responses to drought stress, including, for example, those regulating inhibition of apoptosis and immunity (Table 4).

**Table 4:** Key differentially expressed genes following drought challenge in *P. grandis* and *U. tetralasmus*.

Gene description		NCBI Accession number	*P. grandis*				*U. tetralasmus*			t-test *p*<0.05
			Contig ID	FC		p-value	Contig ID	FC	p-value	
**Chaperone/HSP (** ***p*** ** = 0.0277)**										
**60 kDa heat shock protein**		EKC31862.1	fl_ctg_778	**2.7**		4.7E-05	ph_ctg_636	**2.0**	7.1E-03	
**78 kDa glucose-regulated protein**		EKC33663.1	fl_ctg_1364	**6.0**		3.6E-43	ph_ctg_1443	**3.8**	2.6E-31	
**Alpha-crystallin B chain**		EKC27067.1	fl_ctg_1125	**35.8**		2.8E-11	ph_ctg_1374	**7.2**	1.5E-152	*
**DnaJ-like protein subfamily A member 1**		EKC32228.1	fl_ctg_129	**3.4**		1.3E-02	ph_ctg_1424	**2.2**	1.9E-04	
**DnaJ-like protein subfamily A member 2**		EKC18925.1	fl_ctg_188	**2.0**		2.5E-15	ph_ctg_58	**1.6**	2.2E-05	*
**Endoplasmin**		EKC38233.1	fl_ctg_1292	**3.1**		7.1E-35	ph_ctg_790	**2.1**	1.8E-08	*
**Heat shock 10kDa protein**		NP_001084708.1	fl_ctg_489	**5.3**		4.9E-03	ph_ctg_1163	**2.1**	4.7E-06	*
**Heat shock 70 kDa protein 12A, type 1**		EKC29170.1	fl_ctg_1046	**−2.7**		1.5E-02	ph_ctg_1795	**−5.8**	4.2E-04	
**Heat shock 70 kDa protein 14**		EKC26064.1	fl_ctg_506	**4.6**		4.5E-05	*nde_ctg_5*	***1.3***	*4.5E-01*	*
**Heat shock protein 68**		EKC22243.1	fl_ctg_2557	**24.6**		1.7E-02	ph_ctg_1676	**15.9**	1.2E-06	
**Heat shock protein 70 B2, type2**		EKC30019.1	fl_ctg_1763	**4.61**		0	ph_ctg_1662	**1.66**	4.3E-21	*
**Heat shock protein beta-1, type 2**		EKC40046.1	fl_ctg_683	**21.5**		1.6E-10	ph_ctg_2231	**10.6**	1.8E-243	*
**Heat shock protein beta-1, type 3**		EKC40046.1	fl_ctg_1192	**58.7**		5.0E-07	ph_ctg_1086	**21.3**	9.3E-78	*
**Heat shock protein HSP 90-alpha 1**		EKC25687.1	fl_ctg_2540	**9.5**		5.5E-18	ph_ctg_1044	**9.5**	8.9E-65	
**Stress-70 protein, mitochondrial**		EKC18038.1	fl_ctg_1597	**3.4**		7.0E-03	ph_ctg_1927	**1.6**	3.8E-08	
**BAG molecular chaperone regulator 4**		EKC42633.1	fl_ctg_2336	**27.9**		6.0E-16				
**Calnexin**		EKC32723.1	fl_ctg_224	**6.1**		2.4E-08				
**Calreticulin**		EKC23905.1	fl_ctg_1087	**2.7**		1.1E-16				
**DnaJ-like protein subfamily B member 4**		EKC35183.1	fl_ctg_2051	**6.3**		1.1E-41				
**Heat shock 70 kDa protein 12A, type 2**		EKC19496.1	fl_ctg_2338	**3.6**		2.7E-02				
**Heat shock factor protein 1**		EKC33988.1	fl_ctg_757	**6.1**		2.3E-02				
**Heat shock protein 70 B2, type1**		EKC21713.1	fl_ctg_2493	**8.9**		2.6E-03				
**Heat shock protein beta-1, type 1**		EKC27576.1	fl_ctg_1863	**173.2**		2.9E-10				
**HSPB1-associated protein 1**		EKC31176.1	fl_ctg_1565	**4.0**		1.3E-05				
**Heat shock 70 kDa protein 12B**		EKC29173.1					ph_ctg_1045	**7.4**	3.2E-06	
**Antioxidant/Oxidative Stress Response (p = 0.1534)**										
**Calpain-5**		EKC27521.1	fl_ctg_1914	**1.7**		1.1E-02	ph_ctg_688	**4.4**	1.1E-05	
**Ceruloplasmin**		EKC34134.1	fl_ctg_810	**4.8**		6.8E-31	ph_ctg_1266	**−17.3**	1.6E-02	*
**Dual oxidase**		EKC42615.1	fl_ctg_941	**−7.0**		2.1E-03	ph_ctg_2002	**−4.4**	2.6E-02	
**Glutathione peroxidase 1**		EKC25678.1	fl_ctg_2179	**2.9**		2.6E-14	ph_ctg_1277	**2.2**	9.9E-12	
**Pantetheinase**		EKC31982.1	fl_ctg_1534	**2.6**		1.0E-04	*nde_ctg_11*	***1.2***	*6.5E-01*	
**Protein toll**		EKC33894.1	fl_ctg_829	**10.9**		2.7E-04	ph_ctg_1155	**3.1**	4.9E-02	*
**Selenide, water dikinase**		EKC33185.1	*nde_ctg_4*	***1.3***		*2.4E-02*	ph_ctg_1540	**2.3**	7.7E-20	*
**Selenoprotein M**		NP_001186811.1	fl_ctg_1703	**−3.7**		3.3E-02	ph_ctg_1989	**−2.4**	4.2E-04	
**Selenoprotein P, plasma, 1b**		AAH86844.1	fl_ctg_2541	**−2.1**		1.3E-04	ph_ctg_1412	**−2.1**	0	
**Selenoprotein T**		XP_974477.1	fl_ctg_718	**3.0**		6.9E-10	ph_ctg_1960	**2.0**	3.6E-04	
**Superoxide dismutase**		EKC26875.1	fl_ctg_1390	**−3.2**		1.4E-04	ph_ctg_460	**−3.4**	2.2E-02	
**Thioredoxin reductase 3**		EKC27867.1	fl_ctg_1736	**−1.8**		2.1E-02	ph_ctg_2174	**−2.0**	7.8E-06	
**X-box-binding protein 1**		EKC27673.1	fl_ctg_2520	**33.0**		2.0E-67	ph_ctg_388	**2.5**	1.1E-06	*
**Elongation factor 1-alpha**		EKC33063.1	fl_ctg_1966	**2.5**		1.4E-02				
**Ubiquitin-protein ligase E3C**		EKC38417.1	fl_ctg_1472	**9.8**		1.5E-03				
**Calcium homeostasis endoplasmic reticulum**		EKC18572.1					ph_ctg_2138	**6.1**	5.9E-03	
**Selenoprotein W**		NP_001159799.1					ph_ctg_685	**−1.7**	3.6E-07	
**Cell proliferation/Apoptosis (p = 0.2148)**										
**AP-2 complex subunit mu-1**		EKC19414.1	fl_ctg_415	**−2.0**	2.3E-02		ph_ctg_784	**21.5**	1.6E-05	*
**Apoptosis regulator R1**		EKC20695.1	fl_ctg_2184	**1.7**	6.0E-03		ph_ctg_159	**2.1**	1.7E-02	
**BNIP1**		EKC19480.1	fl_ctg_43	**2.8**	4.1E-02		ph_ctg_164	**11.0**	1.3E-13	*
**BNIP3**		EKC35529.1	fl_ctg_593	**3.8**	1.6E-07		ph_ctg_2228	**3.2**	2.3E-13	
**Bcl-2-like protein 1**		EKC30554.1	fl_ctg_1908	**2.8**	1.7E-27		ph_ctg_493	**3.4**	5.4E-06	
**Cathepsin L-like cysteine proteinase**		EKC36430.1	fl_ctg_2106	**2.3**	3.6E-08		ph_ctg_523	**−2.1**	5.0E-02	*
**Cold shock domain-containing protein E1**		EKC28502.1	fl_ctg_1124	**1.8**	2.3E-10		ph_ctg_2058	**2.0**	2.6E-04	
**Corticosteroid 11-beta-dehydrogenase 2**		EKC40090.1	fl_ctg_1159	**32.2**	1.2E-06		ph_ctg_608	**7.4**	5.0E-02	*
**Cyclin-I**		EKC18711.1	fl_ctg_2271	**2.0**	6.2E-09		ph_ctg_2184	**2.0**	1.7E-05	
**E3 ubiquitin-protein ligase MIB2**		EKC39104.1	fl_ctg_2263	**−6.2**	2.1E-02		ph_ctg_1106	**3.6**	4.6E-05	*
**GADD45 alpha**		EKC31645.1	fl_ctg_62	**28.2**	1.2E-05		*nde_ctg_12*	***−1.2***	*6.3E-01*	*
**Inhibitor of apoptosis protein**		EKC20774.1	fl_ctg_1444	**14.7**	7.8E-24		ph_ctg_1060	**11.9**	6.7E-03	
**Krueppel-like factor 5**		EKC17625.1	fl_ctg_1639	**25.8**	8.1E-60		ph_ctg_1332	**3.7**	3.0E-04	*
**Polycomb complex protein BMI-1**		EKC23428.1	fl_ctg_1135	**2.7**	7.7E-06		ph_ctg_1337	**2.9**	2.0E-08	
**Protein BTG1**		EKC25710.1	fl_ctg_1732	**9.1**	8.4E-175		ph_ctg_167	**1.5**	1.0E-02	*
**Serine/threonine-protein kinase Pim-3**		EKC19760.1	fl_ctg_1493	**3.7**	6.6E-13		ph_ctg_1242	**−2.4**	1.3E-08	*
**Stress-induced-phosphoprotein 1**		EKC18743.1	fl_ctg_1646	**3.5**	3.5E-08		ph_ctg_1651	**2.5**	9.8E-08	*
**TMBIM4**		EKC24718.1	fl_ctg_853	**2.8**	1.3E-02		ph_ctg_2481	**2.0**	3.1E-12	*
**Ubiquitin**		EKC42613.1	fl_ctg_971	**14.0**	5.0E-13		ph_ctg_231	**2.0**	1.0E-31	*
**Antigen KI-67**		EKC25595.1	fl_ctg_1316	**−4.9**	1.3E-02					
**Caspase-10**		EKC25819.1	fl_ctg_1166	**8.2**	2.8E-05					
**CDH1-D**		AAL31950.1	fl_ctg_1990	**2.4**	2.8E-06					
**MAP kinase signal-integrating kinase 1**		EKC18571.1	fl_ctg_1707	**3.9**	1.8E-10					
**Protein SET**		EKC28777.1					ph_ctg_761	**−2.6**	2.7E-04	
**Putative inhibitor of apoptosis**		EKC26950.1					ph_ctg_868	**86.8**	6.5E-26	
**Ubiquitin-conjugating E2 variant 1 (UEV1A)**		EKC31486.1					ph_ctg_910	**4.5**	5.1E-14	
**Energy Metabolism (p = 0.1482)**										
**5′-AMP-activated protein kinase beta-2**		EKC39833.1	fl_ctg_2083	**−4.3**		1.2E-05	ph_ctg_2306	**−2.1**	2.9E-02	*
**ATP synthase F0 subunit 6**		YP_003355006.1	fl_ctg_2323	**−7.1**		5.8E-08	*nde_ctg_6*	***−3.3***	*2.7E-01*	
**Cholecystokinin receptor**		EKC30410.1	fl_ctg_1030	**3.8**		1.1E-04	ph_ctg_337	**−3.3**	4.9E-02	*
**Cytochrome b**		YP_003354999.1	fl_ctg_1821	**−5.6**		0	ph_ctg_589	**−2.7**	2.1E-02	
**Cytochrome c oxidase subunit I**		YP_003354995.1	fl_ctg_1261	**−3.4**		0	ph_ctg_288	**−1.6**	1.3E-13	*
**Cytochrome c oxidase subunit II**		YP_003354996.1	fl_ctg_1138	**−3.3**		0	ph_ctg_23	**−1.9**	3.1E-04	
**Cytochrome c oxidase subunit III**		YP_003355007.1	fl_ctg_1487	**−4.5**		0	*nde_ctg_7*	***−2.4***	*3.1E-01*	
**Glutamine synthetase 2 cytoplasmic**		EKC27195.1	fl_ctg_1817	**5.3**		1.5E-24	ph_ctg_1688	**2.0**	1.6E-24	*
**Group XIIA secretory phospholipase A2**		EKC23468.1	fl_ctg_2457	**2.8**		1.6E-03	ph_ctg_1198	**10.9**	5.0E-05	*
**NADH dehydrogenase subunit 1**		YP_003355001.1	fl_ctg_1537	**−6.0**		0	*nde_ctg_14*	***−1.3***	*8.8E-01*	*
**NADH dehydrogenase subunit 4**		YP_003355003.1	fl_ctg_1864	**−2.0**		1.5E-03	*nde_ctg_8*	***−1.1***	*9.5E-01*	
**NADH dehydrogenase subunit 5**		YP_003355000.1	fl_ctg_1887	**−3.8**		2.5E-12	ph_ctg_503	**1.7**	1.5E-03	*
**Succinyl-CoA ligase**		EKC28914.1	fl_ctg_1283	**−2.2**		2.7E-03	ph_ctg_1091	**−3.5**	6.2E-06	
**ATF5 activating transcription factor 5**		EKC37224.1	fl_ctg_360	**2.4**		9.5E-07				
**Immune (p = 0.7569)**										
**B-cell lymphoma 3-encoded protein**		EKC39819.1	fl_ctg_200	**6.2**		1.2E-02	ph_ctg_1522	**−2.4**	4.0E-06	*
**Histone H4 transcription factor**		EKC36777.1	fl_ctg_2257	**6.0**		5.8E-08	*nde_ctg_9*	***1.9***	*5.5E-01*	*
**Interferon regulatory factor 2**		EKC43156.1	fl_ctg_1121	**−2.5**		1.4E-06	ph_ctg_76	**−4.2**	3.3E-10	
**Interleukin-1 receptor-associated kinase 1BP1**		AER35157.1	*nde_ctg_2*	***1.1***		*6.0E-01*	ph_ctg_1648	**−1.7**	1.3E-02	*
**Lipopolysaccharide-binding protein**		EKC33909.1	fl_ctg_2167	**−2.8**		9.7E-03	ph_ctg_1475	**−2.0**	1.2E-03	
**LPS-induced tumor necrosis factor-alpha**		EKC36231.1	fl_ctg_1784	**−4.3**		2.0E-03	ph_ctg_1823	**−2.3**	4.9E-02	
**Lysozyme**		EKC39289.1	fl_ctg_477	**2.1**		4.1E-02	ph_ctg_2014	**2.3**	3.0E-10	
**NLR family, pyrin domain containing 3**		XP_003730107.1	fl_ctg_758	**4.5**		9.8E-04	ph_ctg_1933	**2.7**	1.5E-03	
**NF-kappa-B inhibitor cactus**		EKC37718.1	fl_ctg_2206	**3.9**		1.9E-18	ph_ctg_935	**2.1**	2.5E-04	*
**Peptidoglycan-recognition protein SC2**		EKC42861.1	fl_ctg_1392	**3.4**		3.0E-03	ph_ctg_1182	**3.4**	1.6E-13	
**Phospholipid scramblase 2, partial**		EKC25020.1	fl_ctg_585	**−4.0**		2.0E-02	ph_ctg_894	**14.3**	5.4E-06	*
**Serine/threonine-protein kinase TBK1**		EKC41453.1	fl_ctg_1373	**7.0**		3.2E-31	ph_ctg_1115	**11.3**	3.4E-03	
**Slit-like protein 1 protein**		EKC33230.1	fl_ctg_1877	**6.5**		7.0E-06	*nde_ctg_13*	***1.2***	*5.2E-01*	*
**TNFAIP3-interacting protein 2**		EKC38434.1	fl_ctg_840	**4.7**		4.3E-04	ph_ctg_1846	**2.5**	2.2E-02	
**TNF receptor-associated factor 2**		EKC31562.1	*nde_ctg_1*	***1.9***		*5.4E-01*	ph_ctg_783	**4.5**	1.7E-03	*
**Toll-like receptor 2 type-2**		EKC18337.1	fl_ctg_1332	**3.8**		9.9E-06	ph_ctg_1237	**4.0**	1.3E-10	
**Toll-like receptor 3**		EKC35956.1	fl_ctg_1369	**−3.0**		3.4E-03	ph_ctg_26	**4.6**	7.1E-03	*
**14-3-3 protein epsilon**		EKC29146.1	fl_ctg_1686	**−4.2**		9.8E-13				
**Myeloid differentiation primary response 88**		EKC40065.1	fl_ctg_2378	**3.6**		3.0E-03				
**Tax1-binding protein 1-like protein B**		EKC20118.1	fl_ctg_2123	**−1.5**		8.1E-03				
**14-3-3 protein zeta**		EKC18419.1					ph_ctg_1953	**−7.8**	1.3E-04	
**Complement C3**		EKC24393.1					ph_ctg_2299	**−1.6**	1.7E-06	
**Defensin**		ADO32580.1					ph_ctg_2420	**100.1**	1.9E-06	
**EIF4EBP1**		EKC38736.1					ph_ctg_889	**2.5**	1.1E-07	
**NLR family, pyrin domain containing 1**		XP_002731351.1					ph_ctg_1301	**13.2**	2.2E-03	
**TNF receptor-associated factor 3**		EKC22057.1					ph_ctg_2244	**3.3**	4.5E-03	
**Toll-like receptor 13**		EKC26527.1					ph_ctg_1671	**−10.7**	1.8E-02	
**Cytoskeletal (p = 0.0149)**										
**Collagen alpha-2(I) chain**		EKC19703.1	fl_ctg_1852	**−2.3**		1.0E-02	ph_ctg_1792	**−1.9**	1.5E-05	
**Fibrillin-1-like**		EKC40135.1	fl_ctg_1999	**−2.3**		2.0E-02	ph_ctg_2292	**−6.5**	1.2E-03	*
**Microtubule-associated protein 2**		EKC35104.1	fl_ctg_1783	**40.1**		4.4E-08	ph_ctg_664	**5.9**	6.1E-04	*
**Myosin catalytic light chain LC-1**		EKC20656.1	*nde_ctg_3*	***1.2***		*8.4E-02*	ph_ctg_1040	**−13.0**	2.1E-04	*
**Protocadherin Fat 4**		EKC27752.1	fl_ctg_2135	**1.9**		8.5E-03	ph_ctg_1385	**−16.5**	2.8E-07	*
**Ras-like GTP-binding protein Rho1**		EKC37733.1	fl_ctg_982	**15.5**		3.8E-03	ph_ctg_997	**−2.7**	4.9E-02	*
**Tubulin alpha-1C chain**		EKC19808.1	fl_ctg_738	**−2.4**		1.3E-10	ph_ctg_1023	**−2.2**	5.4E-09	
**Tubulin beta chain**		EKC30036.1	fl_ctg_408	**33.1**		7.9E-31	ph_ctg_2439	**3.6**	1.2E-03	*
**Dynein heavy chain 6, axonemal**		EKC31674.1	fl_ctg_2151	**−2.8**		7.6E-04				
**Tubulin alpha chain**		EKC22339.1	fl_ctg_442	**−3.4**		3.7E-08				
**Other (p = 0.8545)**										
**Chitin synthase 1**	EKC23809.1		fl_ctg_835	**−3.5**		2.1E-04	ph_ctg_1713	**−4.3**	3.5E-06	
**Chloride intracellular channel exc-4**	EKC30516.1		fl_ctg_1892	**5.2**		6.5E-22	*nde_ctg_10*	***1.4***	*1.8E-02*	*
**Far upstream element-binding protein 3**	NP_001034426.1		fl_ctg_614	**−31.2**		0	ph_ctg_666	**−1.9**	1.8E-02	*
**Fibulin-2**	EKC31789.1		fl_ctg_604	**−3.9**		6.4E-03	ph_ctg_1457	**−3.8**	5.0E-08	
**Hemicentin-1**	EKC33127.1		fl_ctg_1013	**15.8**		4.9E-21	ph_ctg_1370	**−2.4**	0	*
**Krueppel-like factor 11**	EKC40859.1		fl_ctg_1120	**3.9**		4.1E-07	ph_ctg_245	**3.6**	5.0E-02	
**Outer dense fiber protein 3**	NP_001171751.1		fl_ctg_846	**−12.2**		8.1E-04	ph_ctg_803	**4.0**	4.9E-02	*
**Putative accessory gland protein**	ABG01813.1		fl_ctg_498	**21.0**		5.2E-20	ph_ctg_280	**2.2**	4.5E-05	*
**Tribbles-like protein 2**	EKC22747.1		fl_ctg_1325	**10.4**		2.3E-164	ph_ctg_2106	**1.9**	3.0E-09	*
**Water and ammonia transporting aquaporin**	EKC35418.1		fl_ctg_1403	**2.3**		1.5E-04	ph_ctg_1774	**−1.6**	2.4E-05	*
**Epithelial membrane protein 2**	EKC25041.1		fl_ctg_113	**17.2**		3.2E-07				
**Liver stage antigen 3 precursor**	XP_002257838.1		fl_ctg_642	**−19.1**		1.3E-15				
**Semenogelin II precursor**	ABO52944.1		fl_ctg_628	**−16.5**		0				
**Dexras1**	XP_002430253.1						ph_ctg_1269	**24.8**	1.7E-02	

Genes were organized in broad functional categories. Absence of contig, fold change, and p-value information for a given gene indicates that no orthologous sequence was detected in that particular species. Italicized contig identifiers indicate that the gene did not fit criteria for significance (≥1.5 fold change, *p*<0.05) in the particular species. * indicates significantly different fold changes (FC) between species for a given gene based on a t-test of replicated fold change values (*p*<0.05). Differences in fold changes between species in each category were assessed for significance using a paired t-test (*p*<0.05) with values given next to the category titles. Additional information for each contig is available in Table S3.

### Validation of RNA-Seq profiles by QPCR

**Figure 1:**
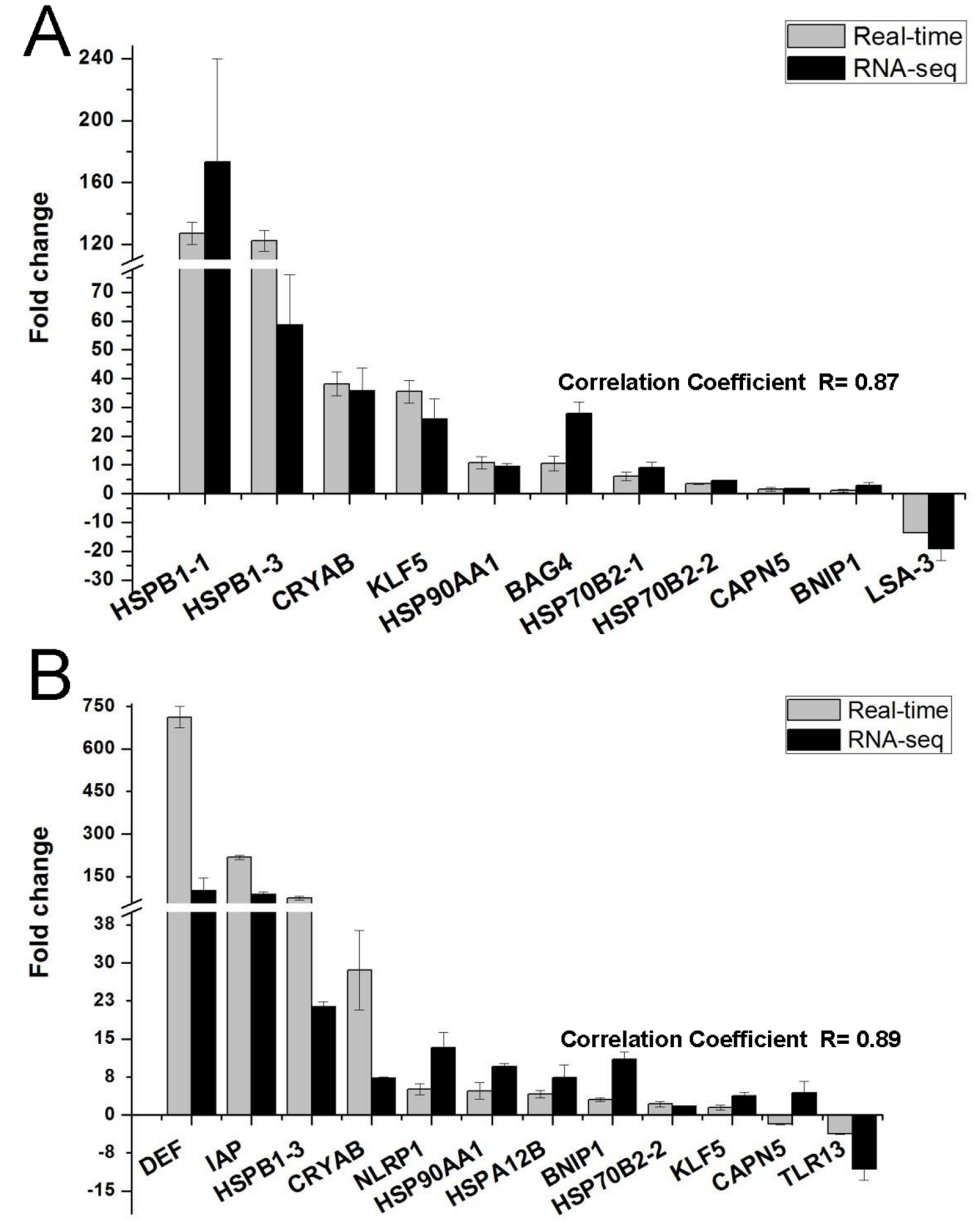
Comparison of fold changes between RNA-Seq and QPCR results in selected genes of *P. grandis* (A) and *U. tetralasmus* (B) at 72 h of heat/desiccation exposure. QPCR fold changes are relative to control samples and normalized by changes in beta-actin values. Gene abbreviations are: Defensin, DEF; Inhibitor of apoptosis protein, IAP; Heat shock protein beta-1, type 3,HSPB1-3; Alpha-crystallin B chain, CRYAB; NLR family, pyrin domain containing 1, NLRP1; Heat shock protein HSP 90-alpha 1, HSP90AA1; heat shock 70 kDa protein 12B-like, HSPA12B; BCL2/adenovirus E1B interacting protein 1-like, BNIP1; Heat shock protein 70 B2, type2, HSP70B2-2; Kruppel-like factor5, KLF5; Calpain 5, CAPN5; Toll-like receptor 13, TLR13; Heat shock protein beta-1, type 1,HSPB1-1; BAG family molecular chaperone regulator 4, BAG4; Heat shock protein 70 B2,type1, HSP70B2-1; Liver stage antigen 3 precursor, LSA-3. Contig IDs are available in Table S1.

In order to validate the differentially expressed genes identified by RNA-Seq, we selected 16 genes for QPCR confirmation from *P. grandis* and *U. tetralasmus* (Table S5). Selection of genes was based on putative functions in response to heat stress/desiccation and pathway analyses. Melting curve analysis revealed a single product for all tested genes. Fold changes from QPCR were compared with the RNA-Seq expression analysis results. QPCR results for *P. grandis* were significantly correlated with RNA-Seq results (avg. correlation coefficient, R = 0.87; p-value <0.001; Figure 1A). Similarly, with the exception of calpain 5, QPCR results for *U. tetralasmus* closely matched those observed by RNA-Seq results (avg. correlation coefficient, R = 0.89; p-value <0.001; Figure 1B). Overall, the QPCR results indicated the reliability and accuracy of the Trinity reference assembly and the RNA-Seq-based transcriptome expression analysis.

### Temporal expression of key heat/desiccation signatures

**Figure 2:**
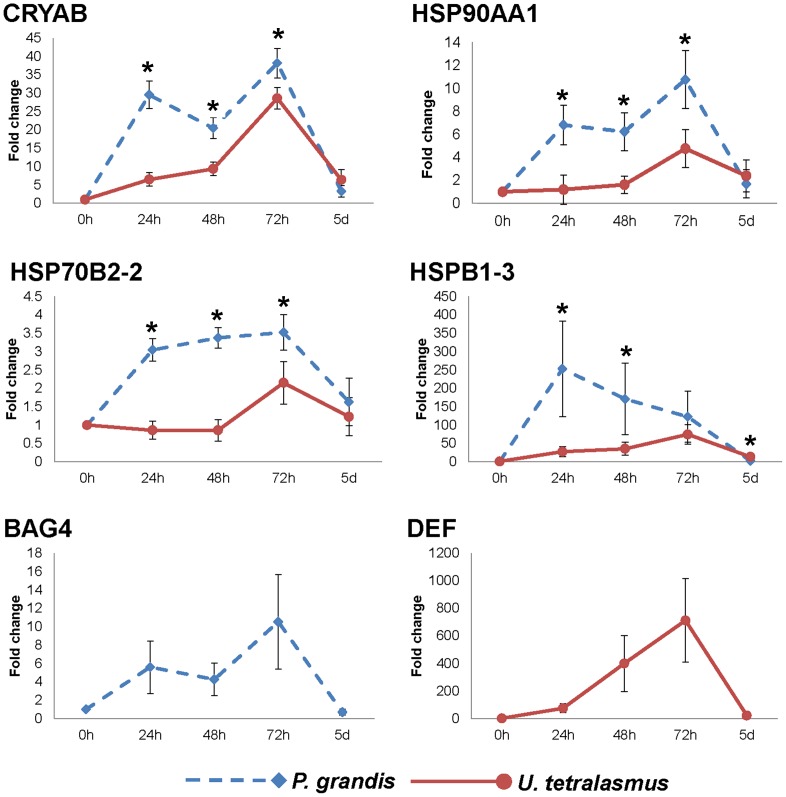
Temporal profiling of key genes by QPCR in *P. grandis* and *U. tetralasmus*. Fold changes are relative to control values (set as 1) and normalized by changes in beta-actin values. * indicates significantly different fold changes between species (p<0.05). Error bars reflect standard error of the mean. Gene abbreviations are: Alpha-crystallin B chain, CRYAB; Heat shock protein HSP 90-alpha 1, HSP90AA1; Heat shock protein 70 B2, type2, HSP70B2-2; Heat shock protein beta-1, type 3,HSPB1-3; BAG family molecular chaperone regulator 4, BAG4; Defensin, DEF. Contig IDs are available in Table S1.

Given our focus on the 72 h timepoint for RNA-Seq expression analysis, we sought to extend the profiles of several key genes to earlier (0 h, 24 h, 48 h) timepoints as well as the recovery phase (5 d). While financial constraints limited our ability to examine these timepoints comprehensively by RNA-seq, we wished to examine whether key genes identified at 72 h could serve as sensitive markers for stress at other timepoints. We selected four heat shock genes that were up-regulated to differing extents in both species and a gene found to be up-regulated uniquely in each species (Figure 2). We found that heat shock gene responses in *P. grandis* showed faster and higher levels of induction than observed in *U. tetralasmus*. All four shared heat shock proteins (CRYAB, HSP90AA1, HSP70B2-2, HSPB1-3) showed significant induction of expression in the more sensitive *P. grandis* by 24 h, while up-regulation of these genes in *U. tetralasmus* at the same timepoint was either relatively modest or not observed. Expression of the four shared genes trended higher throughout the heat and desiccation timepoints, with the exception of HSPB1-3 in *P. grandis* which peaked at 24 h and then declined. Similar patterns of up-regulation were also observed for BAG molecular chaperone regulator 4 (BAG4) in *P. grandis* and defensin (DEF) in *U. tetralasmus*. All examined genes had declined from their 72 h expression levels in the recovery timepoint, with most genes approaching homeostatic (0 h) levels. Fold changes (relative to 0 h) were significantly higher in *P. grandis* than *U. tetralasmus* at most tested timepoints (Figure 2). Based on this data subset, it seems likely that that other key genes identified at 72 h also responded sensitively to heat/desiccation at earlier timepoints and returned to normal levels as experimental stressors were relaxed (recovery period).

## Discussion

Recent studies of mussel behavioral and physiological responses to drought [2], [3] have revealed an array of strategies to maximize survival, growth, and reproduction. Mussel species inhabiting small streams during drought periods can be stranded by receding waters, forcing them to burrow into cooler, wetter sediments or to track waters to deeper pools, when available. These differing behavioral approaches (tracking, burrowing, or tracking then burrowing) likely result in differential fluctuations in population sizes and stream bed distributions among species based on annual variations in the hydrologic cycle. Physiologically, stranded mussels face the simultaneous stressors of increased temperature and tissue desiccation which can, ultimately, impact survival but, more immediately, regulate the evolved avoidance strategies of each species. We sought here to examine the transcriptional regulation of differing tolerances to drought in two mussel species representing physiological and behavioral extremes. *U. tetralasmus* (pondhorn) can live up to 2 years out of water at cool temperatures, while *P. grandis* (floater) can tolerate only a few weeks of desiccation [30]. Recent field and laboratory studies by our group revealed burrowing behavior in *U. tetralasmus* and tracking behavior in *P. grandis* [3]. In this study, we carried out RNA-Seq transcriptome profiling on *P. grandis* and *U. tetralasmus* exposed to experimental conditions simulating those of a drought-induced stranding event, i.e. heat and desiccation.

Prior to initiation of this research, few genetic resources were available for either *P. grandis* or *U. tetralasmus*, with only a handful of mitochondrial sequences currently in public databases. Therefore, prior to examining transcriptional responses in either species, we needed to sequence and assemble high-quality transcriptomes. Use of Trinity [18] provided superior assembly when compared with Trans-ABySS and generated contigs with average lengths greater than 800 bp. Although it is difficult to predict gene numbers based on comparison with other invertebrate species known for high rates of mutation and diversification [31], estimates of gene numbers from the draft genomes of the Pacific oyster (*Crassostrea gigas*) and the pearl oyster (*Pinctada fucata*) are available. These bivalve species encode 28,027 and 23,257 genes, respectively [29], [32], compared with 22,616 and 20,610 predicted unigene annotations in *P. grandis* and *U. tetralasmus*, respectively (Table 2). Based on predicted low rates of sequence conservation across species, we estimate that the *P. grandis* and *U. tetralasmus de novo* assemblies captured at least 70% of the coding genes of each species.

Sampled tissues and pooling strategies inevitably impact the size, scope, and nature of captured transcriptomes and associated downstream expression profiles. Here, we sampled tissues from the foot of individual mussels using a biopsy punch and pooled individual samples to construct replicate pools for RNA-Seq analysis as in previous studies [15], [26]–[28]. While pooling may mask some of the individual variation in homeostatic and induced gene expression profiles, it also serves to normalize outliers and direct focus to key shared responses. Putative biomarkers developed here can be field-validated on individual samples in future studies.

The mussel foot is a tough, extendible muscular organ used primarily for locomotion and, in juveniles, for anchoring the animal to substrate via byssal threads. No reports of gene expression in the foot of unionid mussels are available to-date, although flow regimen has recently been reported to impact byssal thread production in juvenile unionids [33]. In the zebra mussel, *Dreissena polymorpha*, gene and protein expression of the foot organ have been studied in the context of adhesion [34]–[36]. Our primary consideration in choosing to examine the foot transcriptome was to obtain samples rapidly and in a non-lethal manner [37]. Given that 70% of freshwater mussels are currently imperiled [4], future wide-scale assessments of individual or population health in the field will require sampling techniques that are rapid and minimally invasive. We, therefore, wanted to obtain relevant signatures of heat/desiccation stress from a tissue available without sacrifice or extensive manipulation of the organism. Additionally, expression signatures in the foot may reflect the resulting differential behavioral impulses for locomotion, adhesion and/or burrowing. While beyond the scope of the present study, a comprehensive tissue expression atlas of certain representative, non-endangered mussel species should be considered in the future.

Several transcriptomic and proteomic studies in bivalves over the last decade have expanded our understanding of the components of thermal stress responses in these species. The most comprehensive studies have been carried out in marine mussels [10]–[13] and in the Pacific oyster [8], [9], [29]. With the exception of Zhang et al. 2012 [30], other studies have utilized microarray technology, potentially missing rare, previously un-sequenced transcripts. Recently, we conducted a smaller-scale heat stress study in the unionid mussel *Villosa lienosa* utilizing RNA-Seq [15]. Examined together, several key components of bivalve heat stress responses can be seen in these studies. These include changes in molecular chaperones, oxidative stress responses, changes in cell turnover and death, changes in energy metabolism, immune and inflammatory responses, and cytoskeletal reorganization. We observed similar dysregulation of genes involved in these pathways in our datasets from *P. grandis* and *U. tetralasmus* (Table 4). Below we highlight putative functions of selected key genes from these categories which may underlie shared and species-specific responses to drought.

### 

#### Chaperones/Heat Shock Proteins

Up-regulation of molecular chaperones, particularly HSP70 family members, has long been understood as a classic indicator of protein damage due to heat [12], [38]. Heat shock proteins (HSPs) interact with stress-denatured proteins, preventing their aggregation. An array of molecular chaperones was induced in both species by exposure to 72 h of elevated temperatures and desiccation (Table 4). Only one chaperone (HSP70-12A1) was down-regulated in both species. A general pattern of higher expression of shared chaperones in the less-tolerant mussel species was present, with 8 of 15 shared chaperones showing a significantly higher fold increase by *P. grandis* compared to *U. tetralasmus,* but none showing a significantly lower fold increase. For example, a small heat shock protein, HSPB1-type 3 was induced 58.7-fold in *P. grandis* compared with 21.3-fold in *U. tetralasmus*. An additional nine HSP family members were unique to *P. grandis*, including HSPB1-type 1 (173.2-fold). These differences were confirmed in selected HSP genes analyzed by QPCR (Figure 1). Further temporal profiling of four shared molecular chaperones showed that species differences in chaperone expression were not confined to the 72 h timepoint but were also present at 24 h and 48 h. *Uniomerus tetralasmus* HSPs showed limited induction prior to 72 h, while *P. grandis* HSPs were rapidly induced by 24 h. Induction of the HSP response was temporal in nature, however, as expression levels of the tested genes all had returned to baseline levels by 5 d after cessation of the emersion/heat exposure (Figure 2). In previous comparisons of marine mussel species and populations with differing heat tolerances, it was observed that differences in small HSP (HSPB-type) up-regulation were among the most significant signatures of heat tolerance/sensitivity [10]–[13]. There, small HSPs were induced at lower temperatures and/or to higher levels in heat-sensitive mussel groups. Small HSPs, often denoted as HSPBs, are a family of heat shock proteins involved in regulation of apoptosis, oxidative stress, and cytoskeletal regulation [39], [40]. Here also, HSPB family members were highly induced in *P. grandis* relative to *U. tetralasmus* indicating their potential importance in determining drought tolerance in unionid mussels.

#### Cell Proliferation/Apoptosis

Accumulation of oxidative damage can induce a number of pro-apoptotic signaling pathways. Conversely, protective/adaptive mechanisms can seek to block excessive apoptotic signals and stimulate cell proliferation and a return to homeostasis [41], [42]. Many of the components of these processes were observed in the sets of differentially expressed genes from both mussel species (Table 4). However, differential regulation of several genes may indicate crucial differences impacting drought tolerance. Genes needed for protein degradation such as ubiquitin were higher in the sensitive *P. grandis* (14.0-fold up-regulated) than in *U. tetralasmus* (2.0-fold). GADD45, a key indicator of DNA damage, was up-regulated 28.2-fold in *P. grandis* but was not induced in *U. tetralasmus*. GADD45 was similarly up-regulated in *Mytilus galloprovincialis* following exposure to metal salts [43]. Similarly, caspase-10, a component of the execution phase of cell apoptosis was up-regulated 8.2-fold in *P. grandis* but unchanged in *U. tetralasmus*. Interestingly, an inhibitor of apoptosis (IAP) gene showed the second highest up-regulation (86.8-fold) of all differentially expressed genes in the drought-tolerant *U. tetralasmus*. No clear IAP orthologue was identified from *P. grandis*. Lastly, ubiquitin-conjugating enzyme E2 variant 1 (UEV1A) was differentially expressed only in *U. tetralasmus* (Table 4). In mammals, IAP genes interact with UEV1A and other ubiquitin ligases such as MIB2 (also differentially expressed here) to inhibit stress-induced apoptosis and facilitate cell proliferation/survival [44]–[46]. These pathways are mediated through NF-κB activation and, therefore, often dovetail with modulation of components of the innate immune system [47,48_ENREF_50]. Taken together, the sensitive *P. grandis* showed induced pro-apoptotic signaling pathways and indicators of DNA damage, whereas the tolerant species *U. tetralasmus* showed greater inhibition of apoptosis and little indication of DNA damage. These molecular differences are likely an important component of the observed physiological differences in drought tolerance between the two species. Further studies are needed in molluscan species to confirm the conserved function of these components in regulation of cell survival.

#### Immune

One area of concern for drought-exposed mussels is that stress responses induced by emersion and/or rising air/water temperatures will impact immune health increasing susceptibilities to pathogens encountered in their environment [9], [33]. Indeed, we observed the dysregulation of a number of important mediators of immunity in both mussel species (Table 4). These included the down-regulation of innate cytokines such as TNF-α and the down-regulation of pathogen-recognition receptors such as Toll-like receptor 3. Several components of the NF-κB signaling pathway including TRAF2, TRAF3, and TBK1 were either present only in *U. tetralasmus* or were induced to a greater extent, potentially regulating cell survival mechanisms as discussed above.

Of particular interest in the immune category was the strong up-regulation of a *U. tetralasmus* defensin (up 100.1-fold; highest FC among pondhorn genes). An orthologous defensin was not identified among differentially expressed genes in *P. grandis*. Defensins are a family of peptides with antimicrobial activities against a range of bacterial and fungal pathogens. While their traditional immune roles have been characterized in marine mussels and oysters [49_ENREF_51,50], little is known about their functions in freshwater mussel. Recently, however, Xu and Faisal [51] described a defensin from zebra mussel, *D. polymorpha*, with antimicrobial activity. Notably, zebra mussel defensin expression was the highest in the foot of all tested tissues and cell types and expression levels corresponded with status of byssogenesis. The authors there speculated that defensin may be providing biological protection against microbial degradation of byssal threads or may be playing a more fundamental role in byssogenesis. Here, in adult unionids, observed differences in defensin up-regulation may reflect differential use of the foot in burrowing. The burrowing species *U. tetralasmus* may scratch or injure its foot in burrowing, resulting in protective secretion of the antimicrobial defensin. By QPCR analysis, up-regulation of *U. tetralasmus* defensin was even higher than that indicated by RNA-Seq (Figure 1) and expression levels rose with the duration of the challenge (Figure 2). Future studies should examine potential functions of unionid defensins in the context of immune status, burrowing, byssal thread production, and drought tolerance.

#### Cytoskeletal

The buildup of free radicals associated with cellular stress leads to damage of the actin cytoskeleton. Changes to actin filament dynamics impact junctional integrity, cell cycling, and cellular movement. Gene and protein changes in cytoskeletal elements have been reported previously in response to heat stress in marine mussels [11], [12]. In response to heat stress, cells modulate expression of these elements and enhance production of small HSPs to combat these effects [52]. In addition to the higher expression of small HSPs in *P. grandis*, larger fold changes were seen in genes associated with assembly of microtubules, one of the fibers composing the cytoskeleton. For example, tubulin beta chain was up-regulated 33.1-fold in *P. grandis*, but only 3.6-fold in *U. tetralasmus* (Table 4). These differences are another indication of higher levels of cellular stress in *P. grandis*.

#### Other

We noted a handful of additional genes with putative functions outside one of the larger response categories. These included genes with functions in ion transport, reproduction, shell structure, and circadian rhythms (Table 4). Interestingly, in both species drought stress led to reduced expression of chitin synthases, key enzymes involved in synthesis of bivalve shells, indicating potential impacts on growth and health from prolonged drought exposure even in “tolerant” species [53]. Finally, dexras1, a gene which, in mammals, is responsible for transducing signals maintaining circadian rhythms of behavior and physiology [54], was sharply induced in *U. tetralasmus* alone (24.8-fold up-regulation). Given the ability of stranded mussels of this species to endure long periods of emersion, it would be of great interest to determine whether dexras1 is responsible for the biological reprogramming needed for tolerating harsh environmental conditions.

## Conclusions

Freshwater mussels in the southeastern US are imperiled by increasingly frequent drought conditions. Here we examined the molecular underpinnings of differing behavioral and physiological responses to drought in two co-occurring mussel species. RNA-Seq transcriptome profiling successfully captured and assembled high quality transcriptomes for *P. grandis* and *U. tetralasmus* and facilitated comparison of gene expression profiles following heat/desiccation exposure. Shared molecular responses included changes in molecular chaperones, oxidative stress profiles, cell cycling, energy metabolism, immunity, and cytoskeletal rearrangements. Significantly higher induction of molecular chaperones and cytoskeletal elements indicated higher levels of cellular stress in *P. grandis* while our results also highlighted individual genes potentially contributing to drought tolerance in *U. tetralasmus*. From this foundation, future studies should seek to validate putative biomarkers revealed here in individual mussels from *P. grandis* and *U. tetralasmus* collected in field settings, as well as examining their utility in assessing stress levels in a broader array of unionid species.

## Supporting Information

Figure S1 Gene ontology (GO) term categorization and distribution of assembled Trinity contigs encoding genes in *P. grandis* (A) and *U. tetralasmus* (B).
(TIF)

Table S1 Primers used for QPCR validation. Primers are listed in the 5′ to 3′ orientation.
(DOCX)

Table S2 Summary of Illumina expressed short reads production and filtering from P. grandis (A) and U. tetralasmus (B). Paired-end reads were generated on a HiSeq 2000 instrument.
(DOCX)

Table S3 Differentially expressed genes in the foot of heat/desiccation challenged and control *P. grandis* and *U. tetralasmus*. Positive/negative values indicate up-regulation and down-regulation, respectively, in drought treatment group relative to the control group. Included genes showed fold changes of 1.5-fold or greater and corrected p-value<0.05. Annotation is based on the NCBI *Crassostrea gigas* and nr databases.
(XLSX)

Table S4 Summary of GO term enrichment result of significantly expressed genes in P. grandis and U. tetralasmus following drought challenge. P-value≤0.1 was considered significant. Population count is the number of genes associated with the term in the population set. Study count is the number of genes associated with the term in the study set.
(DOCX)

Table S5 Fold change values of QPCR validation as presented in Figure 1.
(DOCX)
