# Supplementary figures and images for: Transcriptomic Profiling of Differential Responses to Drought in Two Freshwater Mussel Species, the Giant Floater Pyganodon grandis and the Pondhorn Uniomerus tetralasmus
Source: PLoS One. 2014 Feb 25;9(2):e89481. doi: 10.1371/journal.pone.0089481 (PMC3934898; doi:10.1371/journal.pone.0089481)

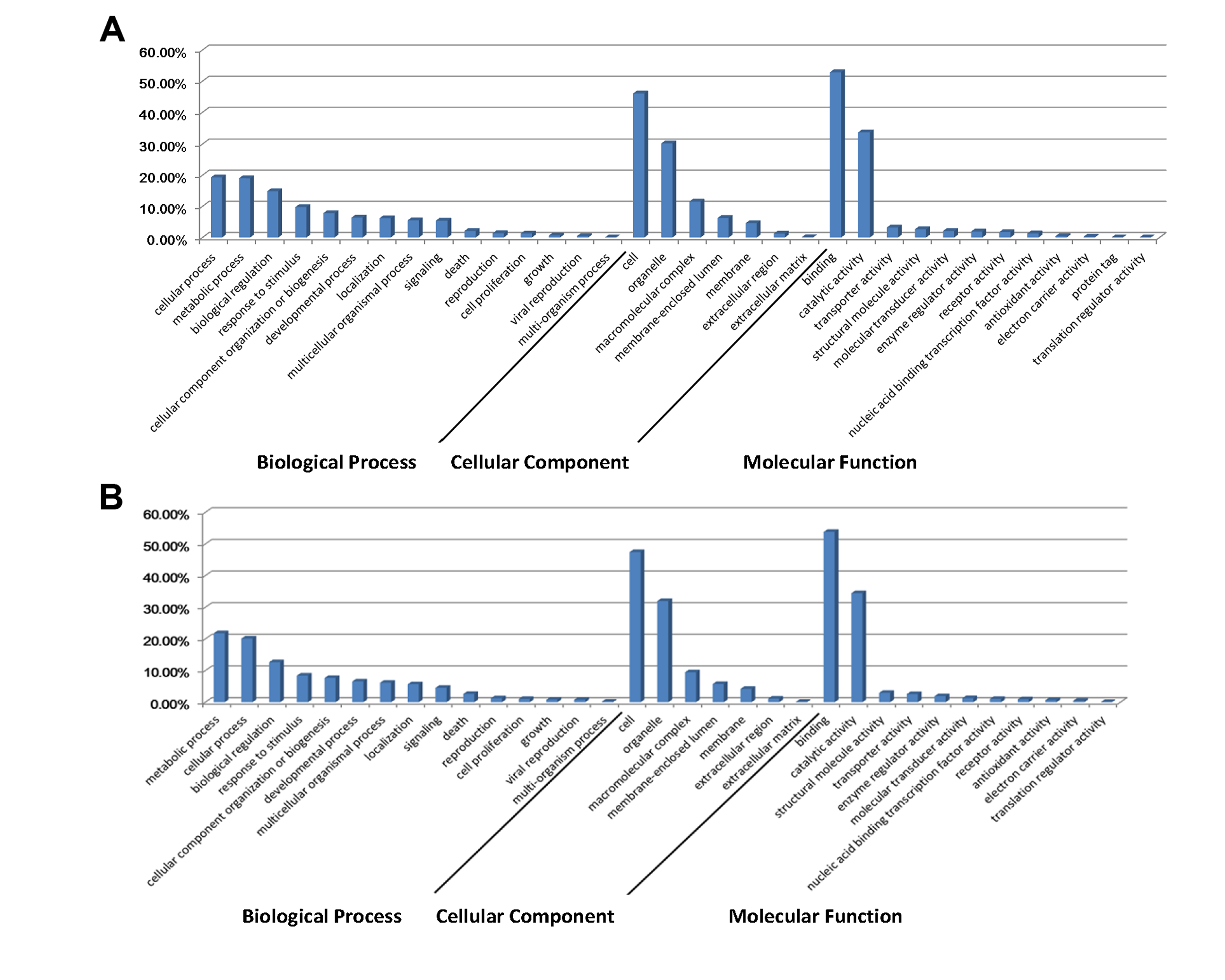

Supplement: Figure S1 — Gene ontology (GO) term categorization and distribution of assembled Trinity contigs encoding genes in P. grandis (A) and U. tetralasmus (B). (TIF) [file pone.0089481.s001.tif]
