# Supplementary material for: Transcriptomic Profiling of Differential Responses to Drought in Two Freshwater Mussel Species, the Giant Floater Pyganodon grandis and the Pondhorn Uniomerus tetralasmus
Source: PLoS One. 2014 Feb 25;9(2):e89481. doi: 10.1371/journal.pone.0089481 (PMC3934898; doi:10.1371/journal.pone.0089481)
Supplement: Table S1 — Primers used for QPCR validation. Primers are listed in the 5′ to 3′ orientation. (DOCX) [file pone.0089481.s002.docx]

**Table S1.** Primers used for QPCR validation. Primers are listed in the 5' to 3' orientation.

| Gene | Contig ID | Forward | Reverse |
| --- | --- | --- | --- |
| *P. grandis* |  |  |  |
| Alpha-crystallin B chain | fl_ctg_1125 | cgcagaatggacagaatg | ttacctccagttcttccg |
| BAG family molecular chaperone regulator 4 | fl_ctg_2336 | aacagcagtcagcgtctca | gttgtggtggtgtcattggt |
| BCL2/adenovirus E1B interacting protein 1 | fl_ctg_43 | tgttagatgctgccttagtc | gaaccatagagaacgcctta |
| Calpain 5 | fl_ctg_1914 | acggaacacagagttatgc | gaggatgaagatgagccaat |
| Heat shock protein 70 B2,type1 | fl_ctg_2493 | gtggatgttgttgtgtctg | gctggaaggtcttgcttat |
| Heat shock protein 70 B2,type2 | fl_ctg_1763 | cctgtctctgtgaatcgtta | gaagaagtctcctcaatggt |
| Heat shock protein beta-1, type 1 | fl_ctg_1863 | gattgataggcacgctgat | gctggaagtaacggctaa |
| Heat shock protein beta-1, type 3 | fl_ctg_1192 | atcgtcactgaggctgat | tgagaggtatcggattgttc |
| Heat shock protein HSP 90-alpha 1 | fl_ctg_2540 | catcatcctcttctccttca | atcttgtcctcctcctctt |
| Kruppel-like factor 5 | fl_ctg_1639 | cgagaaagccaaacaagg | tgtcctcccacaacgaat |
| Liver stage antigen 3 precursor | fl_ctg_642 | ggaactatcagcaggtagaa | cgaactccacagtcttaca |
| Beta actin |  | actctggtgatggtgtga | agcagtggttgtgaagga |
| *U. tetralasmus* |  |  |  |
| Alpha-crystallin B chain | ph_ctg_1374 | cgcagaatggacagaatg | ttacctccagttcttccg |
| BCL2/adenovirus E1B interacting protein 1 | ph_ctg_164 | tctgaacctgactctccat | caagcaataccacctcctt |
| Calpain 5 | ph_ctg_688 | gaacagtgaacacatcttgg | ggctcatcatcatcctctg |
| Defensin | ph_ctg_2420 | gtggtgcttgacgattatg | cgaggtggctattgtgata |
| Heat shock 70 kDa protein 12B-like | ph_ctg_1045 | gtgttagtgcgttgtaggt | ctgttggagtgaggtattgt |
| Heat shock protein 70 B2, type2 | ph_ctg_1662 | gcagacacattgagaatacc | gacacagaccttcactacc |
| Heat shock protein beta-1, type 3 | ph_ctg_1086 | tccttccttgacagtgac | gccatgaacagattgactc |
| Heat shock protein HSP 90-alpha 1 | ph_ctg_1044 | acggagaagtgcttaacag | gatgacgaggacaagaaca |
| Inhibitor of apoptosis protein | ph_ctg_868 | gactggtcacatggatagag | actgttctccgccttcat |
| kruppel-like factor 5 | ph_ctg_1332 | cgagaaagccaaacaagg | tgtcctcccacaacgaat |
| NLR family, pyrin domain containing 1 | ph_ctg_1301 | caggaaggtcaggttaatca | ggtatgtaacggagatgtca |
| Toll-like receptor 13 | ph_ctg_1671 | ccgcataatcctccgtatag | cctgacacatattccgacaa |
| Beta actin |  | tgtgctatgtggctcttg | gctgttgtaggttgtctca |
