# Supplementary material for: Transcriptomic Profiling of Differential Responses to Drought in Two Freshwater Mussel Species, the Giant Floater Pyganodon grandis and the Pondhorn Uniomerus tetralasmus
Source: PLoS One. 2014 Feb 25;9(2):e89481. doi: 10.1371/journal.pone.0089481 (PMC3934898; doi:10.1371/journal.pone.0089481)
Supplement: Table S2 — Summary of Illumina expressed short reads production and filtering from P. grandis (A) and U. tetralasmus (B). Paired-end reads were generated on a HiSeq 2000 instrument. (DOCX) [file pone.0089481.s003.docx]

**Table S2.** Summary of Illumina expressed short reads production and filtering from *P. grandis* (A) and *U. tetralasmus* (B). Paired-end reads were generated on a HiSeq 2000 instrument.

**A)**

| Sample | Reads (x 10^6^) | Avg.length  (bp) | Reads after trimming(x 10^6^) | Percentage kept | Avg. length after trimming(bp) |
| --- | --- | --- | --- | --- | --- |
| Control 1 | 29.2 | 100 | 28.2 | 96.55% | 94.2 |
| Control 2 | 44.7 | 100 | 43.0 | 96.08% | 94.0 |
| Control 3 | 34.0 | 100 | 32.1 | 94.44% | 93.5 |
| Heat 1 | 31.2 | 100 | 30.0 | 95.91% | 94.0 |
| Heat 2 | 29.5 | 100 | 26.6 | 90.24% | 90.6 |
| Heat 3 | 32.5 | 100 | 31.0 | 95.49% | 93.8 |
| Total | 201.1 |  | 190.9 | 94.79% | 93.4 |

**B)**

| Sample | Reads (x 10^6^) | Avg.length  (bp) | Reads after trimming(x 10^6^) | Percentage kept | Avg. length after trimming(bp) |
| --- | --- | --- | --- | --- | --- |
| Control 1 | 38.8 | 100 | 37.1 | 95.40% | 94.0 |
| Control 2 | 38.7 | 100 | 37.2 | 96.09% | 94.0 |
| Control 3 | 45.1 | 100 | 43.2 | 95.87% | 94.0 |
| Heat 1 | 39.9 | 100 | 38.3 | 96.12% | 94.2 |
| Heat 2 | 34.6 | 100 | 31.2 | 90.43% | 90.9 |
| Heat 3 | 29.3 | 100 | 26.5 | 90.57% | 91.1 |
| Total | 226.4 |  | 213.5 | 94.08% | 93.0 |
