# Supplementary material for: Transcriptomic Profiling of Differential Responses to Drought in Two Freshwater Mussel Species, the Giant Floater Pyganodon grandis and the Pondhorn Uniomerus tetralasmus
Source: PLoS One. 2014 Feb 25;9(2):e89481. doi: 10.1371/journal.pone.0089481 (PMC3934898; doi:10.1371/journal.pone.0089481)
Supplement: Table S4 — Summary of GO term enrichment result of significantly expressed genes in P. grandis and U. tetralasmus following drought challenge. P-value≤0.1 was considered significant. Population count is the number of genes associated with the term in the population set. Study count is the number of genes associated with the term in the study set. (DOCX) [file pone.0089481.s005.docx]

**Table S4.** Summary of GO term enrichment result of significantly expressed genes in *P. grandis* and *U. tetralasmus* following drought challenge. p-value≤0.1 was considered significant. Population count is the number of genes associated with the term in the population set. Study count is the number of genes associated with the term in the study set.

| GO ID | GO Name | Population count | Study count | p-Value(FDR) |
| --- | --- | --- | --- | --- |
| *P. grandis* | | | | |
| GO:0005930 | Axoneme | 68 | 33 | 8.140E-05 |
| GO:0044441 | Cilium part | 103 | 41 | 6.600E-04 |
| GO:0048770 | Pigment granule | 66 | 30 | 4.143E-03 |
| GO:0030286 | Dynein complex | 44 | 20 | 4.143E-03 |
| GO:0006457 | Protein folding | 157 | 49 | 2.814E-02 |
| GO:0051082 | Unfolded protein binding | 91 | 32 | 4.171E-02 |
| GO:0031033 | Myosin filament organization | 12 | 8 | 8.194E-02 |
| *U. tetralasmus* | | | | |
| GO:0050792 | Regulation of viral reproduction | 42 | 20 | 8.470E-04 |
| GO:2000243 | Positive regulation of reproductive process | 39 | 19 | 2.076E-03 |
| GO:0006457 | Protein folding | 153 | 51 | 2.594E-03 |
| GO:0048524 | Positive regulation of viral reproduction | 31 | 16 | 5.965E-03 |
| GO:0060548 | Negative regulation of cell death | 296 | 73 | 1.065E-02 |
| GO:0097300 | Programmed necrotic cell death | 8 | 7 | 1.441E-02 |
| GO:0048770 | Pigment granule | 56 | 22 | 2.375E-02 |
| GO:0002764 | Immune response-regulating signaling pathway | 102 | 29 | 2.783E-02 |
| GO:0070265 | Necrotic cell death | 14 | 9 | 4.015E-02 |
| GO:0051346 | Negative regulation of hydrolase activity | 158 | 39 | 4.345E-02 |
| GO:0010941 | Regulation of cell death | 610 | 122 | 4.725E-02 |
| GO:0010466 | Negative regulation of peptidase activity | 103 | 30 | 4.725E-02 |
| GO:0071542 | Dopaminergic neuron differentiation | 4 | 4 | 5.958E-02 |
| GO:0071779 | G1/S transition checkpoint | 31 | 11 | 6.974E-02 |
| GO:0061135 | Endopeptidase regulator activity | 80 | 25 | 6.996E-02 |
| GO:0005761 | Mitochondrial ribosome | 41 | 19 | 7.255E-02 |
| GO:0004857 | Enzyme inhibitor activity | 138 | 36 | 7.444E-02 |
| GO:0009068 | Aspartate family amino acid catabolic process | 16 | 9 | 7.589E-02 |
