# Supplementary material for: Transcriptomic Profiling of Differential Responses to Drought in Two Freshwater Mussel Species, the Giant Floater Pyganodon grandis and the Pondhorn Uniomerus tetralasmus
Source: PLoS One. 2014 Feb 25;9(2):e89481. doi: 10.1371/journal.pone.0089481 (PMC3934898; doi:10.1371/journal.pone.0089481)
Supplement: Table S5 — Fold change values of QPCR validation as presented in Figure 1. (DOCX) [file pone.0089481.s006.docx]

**Table S5.** Fold change values of QPCR validation as presented in Figure 1.

| Gene | 72h fold change after drought | | | | | |
| --- | --- | --- | --- | --- | --- | --- |
|  | ***P. grandis*** | | | ***U. tetralasmus*** | | |
|  | Realtime | RNA-seq |  | | Realtime | RNA-seq |
| Alpha-crystallin B chain | 38.18 | 35.81 |  | | 28.61 | 7.23 |
| BCL2/adenovirus E1B interacting protein 1 | 1.12 | 2.79 |  | | 3.04 | 11.03 |
| Calpain 5 | 1.50 | 1.70 |  | | -1.77 | 4.37 |
| Heat shock protein 70 B2,type2 | 3.52 | 4.61 |  | | 2.15 | 1.66 |
| Heat shock protein beta-1, type 3 | 122.45 | 58.71 |  | | 74.60 | 21.32 |
| Heat shock protein HSP 90-alpha 1 | 10.76 | 9.54 |  | | 4.76 | 9.51 |
| Kruppel-like factor5 | 35.45 | 25.83 |  | | 1.48 | 3.74 |
| BAG family molecular chaperone regulator 4 | 10.54 | 27.88 |  | |  |  |
| Heat shock protein 70 B2,type1 | 6.06 | 8.93 |  | |  |  |
| Heat shock protein beta-1, type 1 | 127.18 | 173.23 |  | |  |  |
| Liver stage antigen 3 precursor | -13.58 | -19.10 |  | |  |  |
| Defensin |  |  |  | | 712.55 | 100.10 |
| Heat shock 70 kDa protein 12B-like |  |  |  | | 4.15 | 7.39 |
| Inhibitor of apoptosis protein |  |  |  | | 217.02 | 86.77 |
| NLR family, pyrin domain containing 1 |  |  |  | | 5.08 | 13.21 |
| Toll-like receptor 13 |  |  |  | | -3.70 | -10.65 |
| Correlation between Methods | 0.87 | |  | | 0.89 | |
